# Effectiveness of ICI-ICI versus ICI-TKI combinations in patients with IMDC intermediate- and poor-risk metastatic renal cell carcinoma: a sub-analysis of the MEET-URO 33 study

**DOI:** 10.1007/s00262-026-04318-x

**Published:** 2026-02-03

**Authors:** Michele Maffezzoli, Alessio Signori, Alessandro Acunzo, Sebastiano Buti, Michela Bosoni, Elena Verzoni, Andrea Di Marco, Cristian Lolli, Marilena Di Napoli, Martina Fanelli, Alberto Dalla Volta, Cristina Masini, Giandomenico Roviello, Roberto Iacovelli, Alessia Mennitto, Roberto Filippi, Mariella Sorarù, Luigi Formisano, Annalisa Guida, Emanuela Fantinel, Carlo Messina, Lucia Bonomi, Sarah Scagliarini, Cecilia Nasso, Silvia Chiellino, Brigida Anna Maiorano, Filippo Deppieri, Alessia Cavo, Vincenza Conteduca, Silvia Zai, Paolo Andrea Zucali, Marcello Tucci, Francesca Vignani, Francesca La Russa, Laura Lombardo, Claudia Caserta, Federico Paolieri, Francesca Bertolotti, Pasquale Rescigno, Giuseppe Luigi Banna, Giuseppe Fornarini, Davide Bimbatti, Sara Elena Rebuzzi

**Affiliations:** 1 Medicine and Surgery Department, University of Parma, Parma, Italy; 2 Medical Oncology Unit, University Hospital of Parma, Parma, Italy; 3 Department of Health Sciences, Section of Biostatistics, University of Genova, Genoa, Italy; 4 Oncologia Medica Genitourinaria, Fondazione IRCCS Istituto Nazionale dei Tumori, Milan, Italy; 5 Oncology 1 Unit, Istituto Oncologico Veneto IOV - IRCCS, Padua, Italy; 6 Department of Medical Oncology, IRCCS Istituto Romagnolo per lo Studio dei Tumori (IRST) “Dino Amadori”, Meldola, Italy; 7 Department of Urology and Gynecology, Istituto Nazionale Tumori IRCCS Fondazione G. Pascale, Naples, Italy; 8 Department of Medicine (DAME), University of Udine, Udine, Italy; 9 Unit of Medical Oncology, Department of Medical and Surgical Specialties, Radiological Sciences, and Public Health, ASST Spedali Civili di Brescia, University of Brescia, Brescia, Italy; 10 Medical Oncology Unit, AUSL-IRCCS of Reggio Emilia, Reggio Emilia, Italy; 11 Department of Health Sciences, Section of Clinical Pharmacology and Oncology, University of Firenze, Florence, Italy; 12 Facoltà di Medicina e Chirurgia, Rome, Italy; 13 Medical Oncology, Università Cattolica del Sacro Cuore, Fondazione Policlinico, Universitario Agostino Gemelli IRCCS, Rome, Italy; 14 Department of Medical Oncology, Azienda Ospedaliera Universitaria Maggiore Della Carità, Novara, Italy; 15 Medical Oncology 1U, Azienda Ospedaliero-Universitaria Città della Salute e della Scienza di Torino, Turin, Italy; 16 U.O. Oncologia, Ospedale di Camposampiero, Camposampiero, Italy; 17 Division of Pharmacology, Department of Neuroscience, Reproductive and Dentistry Sciences, School of Medicine, Federico II University of Naples, Naples, Italy; 18 Medical Oncology Unit, Santa Maria Hospital, Terni, Italy; 19 Department of Oncology, Azienda Ospedaliera Universitaria Integrata di Verona, University of Verona, Verona, Italy; 20 Clinical Oncology, Ospedale Arnas Civico, Palermo, Italy; 21 Medical Oncology Unit, ASST Papa Giovanni XXIII, Bergamo, Italy; 22 Department of Medical Oncology, AORN “A. Cardarelli”, Naples, Italy; 23 Medical Oncology, Ospedale Santa Corona, Pietra Ligure, Italy; 24 Medical Oncology Unit, IRCCS Policlinico San Matteo, Pavia, Italy; 25 Department of Medical Oncology, IRCCS San Raffaele Hospital, Milan, Italy; 26 Medical Oncology Unit, AULSS 3 Serenissima, Mestre-Venice, Italy; 27 Oncology Unit, ASL3- Villa Scassi Hospital, Genoa, Italy; 28 nit of Medical Oncology and Biomolecular Therapy and CREATE Center for Research and Innovation in Medicine, Department of Medical and Surgical Sciences, University of Foggia, Policlinico Riuniti, Foggia, Italy; 29 Medical Oncology Unit, Azienda Ospedaliera SS. Antonio e Biagio e Cesare Arrigo, Alessandria, Italy; 30 Department of Biomedical Sciences, Department of Oncology, Humanitas University, Pieve Emanuele, Milan Italy; 31 IRCCS, Humanitascxs Clinical and Research Center, Rozzano, Milano Italy; 32 Department of Medical Oncology, Cardinal Massaia Hospital, Asti, Italy; 33 Division of Medical Oncology, Ordine Mauriziano Hospital, Turin, Italy; 34 Ospedale San Bortolo, Ulss 8 Berica, Vicenza, Italy; 35 Oncology Unit, Deptartment of Onco-Ematology, Ramazzini Hospital, Carpi, Ausl Modena Italy; 36 Medical Oncology Unit, Santa Maria Della Misericordia Hospital, Perugia, Italy; 37 Department of Oncology, Hospital of Prato, Azienda USL Toscana Centro, Prato, Italy; 38 Medical Oncology Unit, Ospedale San Paolo, Savona, Italy; 39 Department of Oncology, Candiolo Cancer Institute FPO-IRCCS, Turin, Italy; 40 Centre for Cancer, Translational and Clinical Research Institute, Newcastle University, Newcastle Upon Tyne, UK; 41 Department of Oncology, Portsmouth, Univeristy Hospitals NHS Trust, Portsmouth, UK; 42 Faculty of Science & Health, School of Pharmacy & Biomedical Sciences, University of Portsmouth, Portsmouth, UK; 43 Medical Oncology Unit 1, IRCCS Ospedale Policlinico San Martino, Genoa, Italy; 44 Medical Oncology Unit 2, Azienda Ospedaliero-Universitaria Città della Salute e Della Scienza di Torino, Ospedale Molinette, Turin, Italy

**Keywords:** Metastatic renal cell carcinoma, Real-world, ICI, TKI, Intermediate, Poor

## Abstract

**Background:**

Immune checkpoint inhibitor doublet (ICI-ICI) and ICI plus tyrosine kinase inhibitor (ICI-TKI) regimens are the cornerstone of treatment for metastatic renal cell carcinoma (mRCC), although no head-to-head comparisons are currently available. This study aimed to compare the real-world effectiveness of ICI-ICI versus ICI-TKI combinations in patients with intermediate- and poor-risk mRCC according to International Metastatic RCC Database Consortium (IMDC).

**Methods:**

The Meet-URO 33 study is a multicentre retrospective-prospective registry collecting real-world data on patients with mRCC. Multivariable logistic and Cox models were built for objective response rate (ORR), PFS and OS, with a propensity score (PS) adjustment for baseline imbalances.

**Results:**

Among 1497 patients, 755 were intermediate-risk (199 ICI-ICI, 556 ICI-TKI) and 312 poor-risk (77 ICI-ICI, 212 ICI-TKI). Median follow-up was 14.2 months (8.0 months and 14.5 months in poor- and intermediate-risk subgroups, respectively). In poor-risk patients, median OS was 20.3 versus 12.9 months (HR 0.87, 95% CI 0.59–1.28, *p* = 0.49), and median PFS was 6.7 versus 8.7 months (HR 1.10, 95% CI 0.79–1.54, *p* = 0.53), for ICI-ICI versus ICI–TKI, respectively. In the intermediate-risk patients treated with ICI-ICI versus ICI-TKI, median OS was 37.8 versus 35.5 months (HR 1.08; 95% CI 0.77–1.50; *p* = 0.65), and median PFS was 17.8 versus 18.6 months (HR 1.29, 95% CI 1.00–1.66, *p* = 0.050). ORR was 42.9% versus 45.8% in poor-risk patients (OR 0.72, 95% CI 0.39–1.34, *p* = 0.303) and 48.1% versus 54.3% in intermediate-risk patients (OR 0.71, 95% CI 0.48–1.04, *p* = 0.075).

**Conclusions:**

No statistically significant differences in survival or response were observed between ICI-ICI and ICI-TKI combinations in patients with IMDC intermediate- and poor-risk mRCC.

**Supplementary Information:**

The online version contains supplementary material available at 10.1007/s00262-026-04318-x.

## Introduction

Immune checkpoint inhibitors (ICI) and tyrosine kinase inhibitors targeting angiogenesis (TKIs) are the cornerstones of treatment for patients with metastatic renal cell carcinoma (mRCC) [1, 2]. At present, one combination of ICI-ICI and several combinations of an ICI plus a TKI (ICI-TKI) have demonstrated survival benefit compared to the historical control arm with sunitinib [3–7]. Namely, nivolumab plus ipilimumab (ICI-ICI) has been approved for the intermediate- and poor-risk groups according to the International Metastatic RCC Database Consortium (IMDC), while pembrolizumab plus axitinib, nivolumab plus cabozantinib, pembrolizumab and Lenvatinib, avelumab plus axitinib and toripalimab plus axitinib have been approved regardless of the IMDC risk group [1, 2].

However, head-to-head prospective comparison between ICI-ICI and ICI-TKI are lacking, and treatment choice is based on different patient- and tumor-related characteristics, including IMDC risk group, comorbidity, specific toxicities of TKIs and ICIs, symptomatic or indolent disease, presence of sarcomatoid features [8]. Given the various therapeutic options available, the identification of clinical and biological prognostic and predictive factors able to select patients who will benefit most from a specific treatment is, therefore, a crucial point of current clinical research [9].

The MEET-URO 33 study has been designed to collect multicentre retrospective-prospective observational real-world data on patients with mRCC treated with first-line TKI or immune-combinations to identify potential prognostic and/or predictive factors that can help guiding therapeutic decisions [10]. Specifically, the aim of this sub-analysis was to compare the real-world effectiveness of ICI-ICI versus ICI + TKI in patients with mRCC and IMDC intermediate- and poor-risk.

## Material and methods

### Patients’ population

Meet-URO 33 study is a prospective-retrospective multicentre (51 centres in Italy) observational real-world study, which is currently collecting data from patients aged ≥ 18 years old with a histologically confirmed diagnosis of RCC, and metastatic disease, treated with standard first-line treatments according to clinical practice. A retrospective cohort of patients who received a first-line oncological treatment from January 2021 has also been included. Patients who underwent active surveillance alone or for whom clinical data were not available, were excluded. For the purpose of this analysis, intermediate- and poor-risk patients treated with TKI monotherapy were also excluded, Supplementary Fig. 1.

Ethical committee approval was obtained from the coordinating centre (Istituto Oncologico Veneto, IOV–IRCCS of Padua (Registration number: CESC IOV 2023-78, on 01/06/2023). Documented approval from the Local Ethics Committee of each participating centre was required before study activation at each site.

For this sub-analysis, we extracted demographics, clinical and pathological data, including age, gender, tumor grade and histology, nephrectomy, sites of metastases, type of immune-combination, IMDC risk group, comorbidity, response to therapy and survival outcomes. Imaging scans and laboratory tests were performed according to standard local procedures. Primary objective of this sub-analysis was to compare the real-world effectiveness of ICI-ICI versus ICI+TKI in patients with mRCC and IMDC intermediate- and poor-risk in terms of progression-free survival (PFS), overall survival (OS) and objective response rate (ORR).

### Statistical methods

Baseline characteristics were compared between treatment groups (ICI-ICI vs. ICI-TKI) separately for patients with poor and intermediate IMDC risk using appropriate statistical tests: the chi-square or Fisher’s exact test for categorical variables and the Student’s t-test or Mann–Whitney U test for continuous variables, as appropriate.

PFS was defined as the time from the beginning of the treatment and the progression of the disease or death, whichever occurred first. The OS was intended as the time between treatment initiation and death for any reason. ORR was based on RECIST 1.1 and analysed as a binary endpoint using univariable and multivariable logistic regression models [11]. For time-to-event endpoints—OS and PFS—Kaplan–Meier curves were generated, and differences between groups were assessed using the log-rank test. Hazard ratios (HRs) and 95% confidence intervals (CIs) were estimated using Cox proportional hazards models. The proportional hazards assumption was tested and confirmed.

For each risk group, univariable Cox (for PFS and OS) and logistic (for ORR) were followed by multivariable models adjusting for baseline variables with *p* < 0.15 in univariable comparisons. From this first multivariable analysis were selected variables with a *p* < 0.20 to obtain the final multivariable analysis. The possible differential effect of type of treatment (ICI-ICI vs. ICI-TKI), both in terms of OS and PFS, in the poor- and intermediate-risk group was assessed by using the interaction test. A separate analysis using propensity score adjustment was also conducted, where the propensity score was calculated using a logistic regression model including all covariates associated with treatment choice (ICI-ICI vs. ICI-TKI) with *p* < 0.10. This score was then entered as a covariate in a Cox or logistic model.

To further assess the robustness of our findings and to address potential heterogeneity related to tumor histology, we performed post-hoc sensitivity analyses restricted to patients with clear cell renal cell carcinoma.

All statistical analyses were performed using Stata version 19.0, and a two-sided p-value < 0.05 was considered statistically significant.

## Results

At the time of analysis, a total of 1560 patients were enrolled, though only 1497 were included in the final analysis. Among them, 308 (19.9%) were IMDC good-risk, while 877 (56.5%) and 312 (20.1%) patients were IMDC intermediate- and poor-risk, respectively (Supplementary Fig. 1). Among the 755 intermediate-risk patients, 529 (70.1%) were included retrospectively and 226 (29.9%) prospectively, whereas among the 272 poor-risk patients, 204 (70.6%) were retrospective and 85 (29.4%) prospective.

The median follow-up time was 14.2 months (IQR 6.2–26.0) for the overall population. The detailed list of baseline characteristics for the overall population is available in Supplementary Table 1, while a concise summary including key clinical features is presented in Table 1.

**Table 1:** Summary of key baseline characteristics according to IMDC group

		Poor-risk				Intermediate-risk			
Variable	Category	Total n = 289 n (%)	ICI-ICI n = 77n (%)	ICI-TKI n = 212 n (%)	*p*-value	Total n = 755 n (%)	ICI-ICI n = 199 n (%)	ICI-TKI n = 556 n (%)	*p*-value
Age mean (SD)		64.3 (10.8)	66.4 (11.4)	63.5 (10.5)	0.056		65.7 (10.3)	64.4 (10.8)	0.14
Gender	Females	82 (28.4)	22 (28.6)	60 (28.3)	0.99	196/754 (26.0)	47 (23.6)	149/555 (26.8)	0.37
	Males	207 (71.6)	55 (71.4)	152 (71.7)		558 (73.9)	152 (76.4)	406 (73.2)	
ECOG PS	0	101 (34.9)	29 (37.7)	72 (34.0)	0.83	513/754 (68.0)	144 (72.4)	369 (66.5)	0.33
	1	121 (41.9)	29 (37.7)	92 (43.4)		211 (28.0)	50 (25.1)	161 (29.0)	
	2	58 (20.1)	16 (20.8)	42 (19.8)		27 (3.6)	4 (2.0)	23 (4.1)	
	3	9 (3.1)	3 (3.9)	6 (2.8)		3 (0.4)	1 (0.5)	2 (0.4)	
Cardiovascular comorbidity	Presence	177 (61.2)	58 (75.3)	119 (56.1)	**0.004**	473 (62.7)	132 (66.3)	341 (61.3)	0.21
Genitourinary comorbidity	Presence	31 (10.7)	15 (19.5)	16 (7.5)	**0.008**	110 (14.6)	31 (15.6)	79 (14.2)	0.64
Metabolic or endocrine comorbidity	Presence	84 (29.1)	28 (36.4)	56 (26.4)	0.11	246 (32.6)	84 (42.2)	162 (29.1)	**0.001**
Concomitant drugs	Yes	232/288 (80.6)	68 (88.3)	164/211 (77.7)	**0.045**	571 (75.6)	157 (78.9)	414 (74.5)	0.352
Surgery	Yes	106 (36.7)	31 (40.3)	75 (35.4)	0.49	456/752 (60.6)	136/198 (68.7)	320/554 (57.8)	**0.007**
Histology	Clear cell	232 (80.3)	62 (80.5)	170 (80.2)	0.844	641 (84.9)	179 (89.9)	462 (83.1)	**0.024**
	Chromophobe	3 (1.0)	0 (0.0)	3 (1.4)		11 (1.5)	1 (0.5)	10 (1.8)	
	Poorly differentiated	26 (9.0)	8 (10.4)	18 (8.5)		20 (2.6)	7 (3.5)	13 (2.3)	
	Papillary	15 (5.2)	4 (5.2)	11 (5.2)		47 (6.2)	4 (2.0)	43 (7.7)	
	Other	13 (4.5)	3 (3.9)	10 (4.7)		31 (4.1)	7 (3.5)	24 (4.3)	
Sarcomatoid features	Yes	47/287 (16.4)	22 (28.6)	25/210 (11.9)	**0.001**	94/749 (12.5)	38/197 (19.1)	56/552 (10.1)	**0.001**
Rhabdoid features	Yes	22/287 (7.7)	10 (13.0)	12/210 (5.7)	**0.012**	60/747 (7.9)	24/197 (12.1)	36/550 (6.5)	**0.011**
Necrosis	Yes	82/215 (38.1)	25/61 (41.0)	57/154 (37.0)	0.641	185 (24.5)	62 (31.2)	123 (22.1)	**0.042**
Lung metastases	Presence	194 (67.1)	49 (63.6)	145 (68.4)	0.48	422 (55.9)	123 (61.8)	299 (53.8)	**0.050**
Liver metastases	Presence	55 (19.0)	13 (16.9)	42 (19.8)	0.62	104 (13.8)	13 (6.5)	91 (16.4)	**0.001**
Bone metastases	Presence	131 (45.3)	22 (28.6)	109 (51.4)	**0.001**	232 (30.7)	47 (23.6)	185 (33.3)	**0.011**
Soft tissue metastases	Presence	26 (9.0)	12 (15.6)	14 (6.6)	**0.033**	61 (8.1)	9 (4.5)	52 (9.4)	**0.032**
2nd-line therapy	No	204/267 (76.4)	49/70 (70.0)	155/197 (78.7)	0.14	562 (74.4)	133 (66.8)	429 (77.2)	**0.004**
	Yes	63/267 (23.6)	21/70 (30.0)	42/197 (21.3)		193 (25.6)	66 (33.2)	127 (22.8)	
Meet-Uro score	2.0	169/259 (65.3)	52/70 (74.3)	117/189 (61.9)	0.078	284 (37.6)	83 (41.7)	201 (36.2)	0.075
	3.0	90/259 (34.7)	18/70 (25.7)	72/189 (38.1)		243 (32.2)	68 (34.2)	175 (31.5)	
	4.0	–	–	–		110 (14.6)	20 (10.1)	90 (16.2)	

Bold indicates statistically significant values

TKI: tyrosine-kinase inhibitors; ICI: Immune-checkpoint inhibitor; ECOG: Eastern cooperative oncology group; IMDC: International metastatic RCC database consortium

### IMDC poor-risk

#### Baseline characteristics

Among the poor-risk subgroup, 22 (7.0%) patients were treated with TKI monotherapy, although they were excluded from the final analysis, 77 (24.8%) were treated with ICI-ICI and 212 (68.2%) were treated with ICI-TKI combinations. Axitinib plus pembrolizumab was the most used combination (87, 28.0%) followed by cabozantinib and nivolumab (85, 27.3%) and ipilimumab plus nivolumab (77, 24.8%), Supplementary Table 2.

The men age was 64.3 years (SD 10.8), and most patients were male (71.6%). Most patients had a good performance status, with 76.8% presenting ECOG PS 0 or 1. Regarding histology, the prevalent subtype was clear cell (80.3%), followed by poorly differentiated (9.0%) and papillary (5.2%). A prior surgery had been performed in only 36.7% of patients. Overall, 76.4% of patients did not receive any further treatment upon progression, Table 1.

Patients treated with ICI-ICI combinations were more likely to have sarcomatoid (28.6% vs. 11.9%, *p* = 0.001) and rhabdoid features (13.0% vs. 5.7%, *p* = 0.012), genitourinary (19.5% vs. 7.5%, *p* = 0.008) and cardiovascular (75.3% vs. 56.1%, *p* = 0.004) comorbidities, and were more likely to receive concomitant medications (88.3% vs. 77.7%, *p* = 0.045). Patients receiving ICI-TKI combinations presented more frequently with bone metastases (51.4% vs. 28.6%, *p* = 0.001), Table 1.

#### Survival outcomes by treatment type

Median follow-up was 8.0 months (IQR 3.8–16.7) for the poor-risk subgroup. Among the 311 poor-risk patients, OS was evaluable in 293 patients (94.2%), including 73 treated with ICI-ICI and 202 with ICI-TKI combinations.

**Fig. 1:**
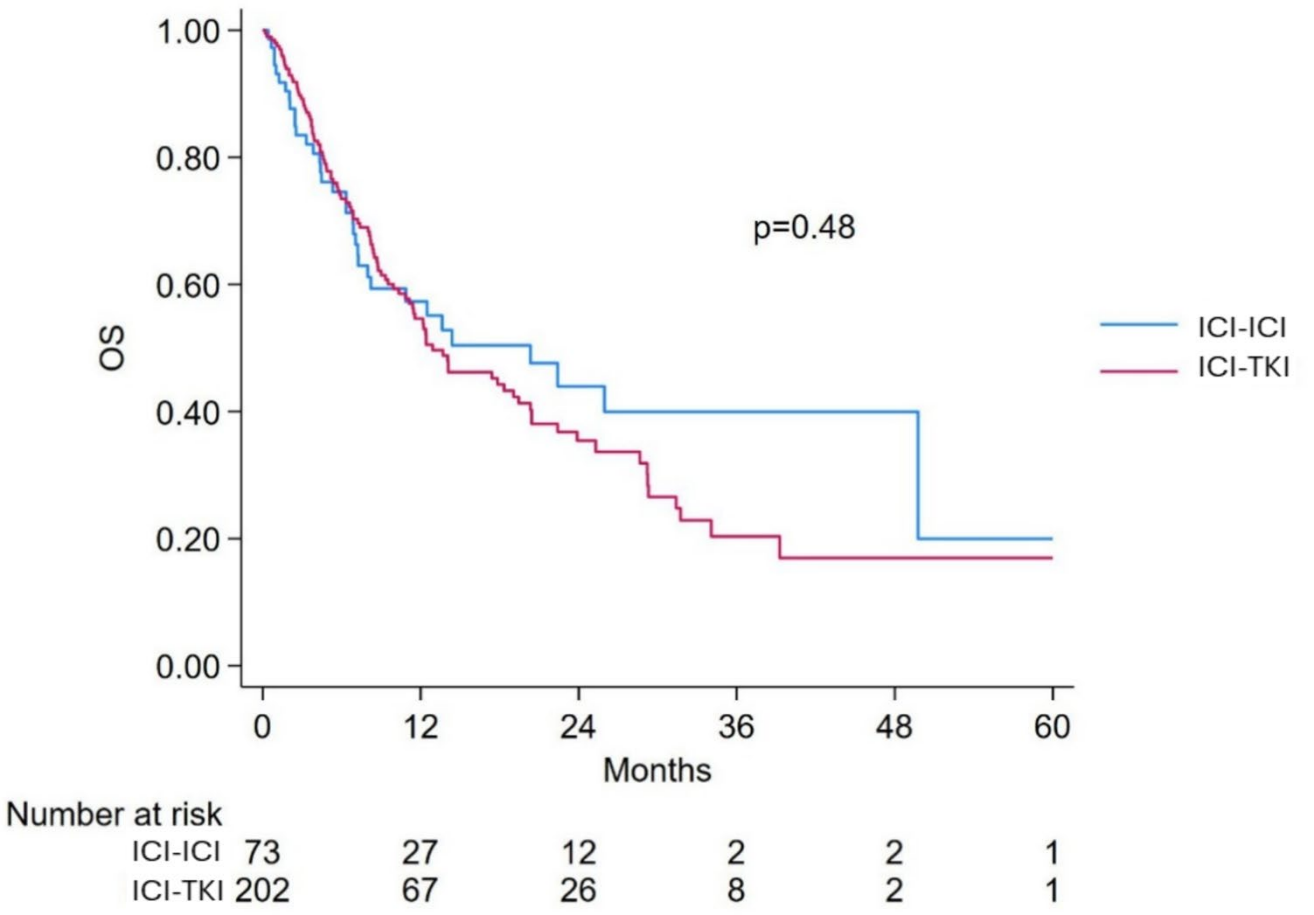
Overall survival in IMDC poor-risk patients treated with ICI-ICI versus ICI-TKI combinations. ICI: immune-checkpoint inhibitors; TKI: tyrosine-kinase inhibitors; OS: Overall survival

The median OS in poor-risk patients was 13.7 months (95%CI 11.1–19.6), with a numerically longer survival in patients treated with ICI-ICI (20.3 months, 95% CI 8.0–NR) compared to those treated with ICI-TKI (12.9 months, 95% CI 10.8–19.4), Fig. 1.

**Fig. 2:**
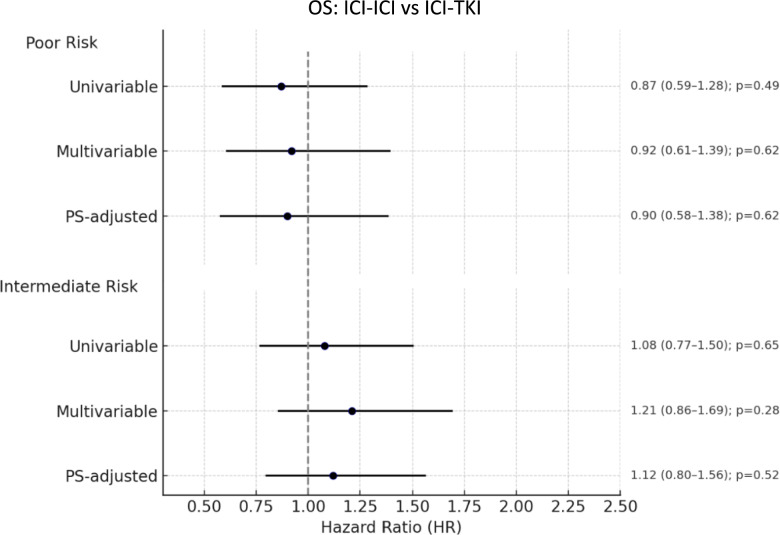
Forest plot of overall survival comparing ICI-ICI versus ICI-TKI combinations in IMDC poor- and intermediate-risk groups. ICI: immune-checkpoint inhibitors; TKI: tyrosine-kinase inhibitors; OS: Overall survival; PS: propensity score

In the univariable analysis, the type of first-line treatment (ICI-ICI vs. ICI-TKI) was not significantly associated with OS (HR 0.87, 95% CI 0.59–1.28, *p* = 0.49), Fig. 2. In the multivariable model, a significant increased risk of death was found to be associated with presence of bone metastases (HR 1.54, 95% CI 1.09–2.18, *p* = 0.014). Patients receiving any concomitant medications had a significantly lower risk of death compared to those without (HR 0.64, 95% CI 0.42–0.99, *p* = 0.044), Table 2. Type of treatment (ICI-ICI vs. ICI-TKI) was not significantly associated with OS (HR 0.92, 95% CI 0.61–1.39, *p* = 0.684), even when the PS was included in the Cox model (HR 0.90, 95% CI 0.58–1.38, *p* = 0.624), Fig. 2.

**Table 2:** Multivariable cox regression analysis for overall survival and progression-free survival in IMDC poor-risk patients

Variable	References	Overall survival		Progression-free survival	
		HR (95% CI)	*p*-value	HR (95% CI)	*p*-value
Treatment ICI-ICI	ICI-TKI	0.92 (0.61–1.39)	0.684	1.18 (0.82–1.69)	0.370
Age	Per year	1.01 (1.00–1.03)	0.103	–	–
Concomitant medications	No	0.64 (0.42–0.99)	**0.044**	0.73 (0.50–1.07)	0.100
Bone metastases	No	1.54 (1.09–2.18)	**0.014**	1.53 (1.12–2.10)	**0.008**
Sarcomatoid component	No	1.65 (0.97–2.46)	0.065	1.58 (1.04–2.38)	**0.031**

Bold indicates statistically significant values

**Fig. 3:**
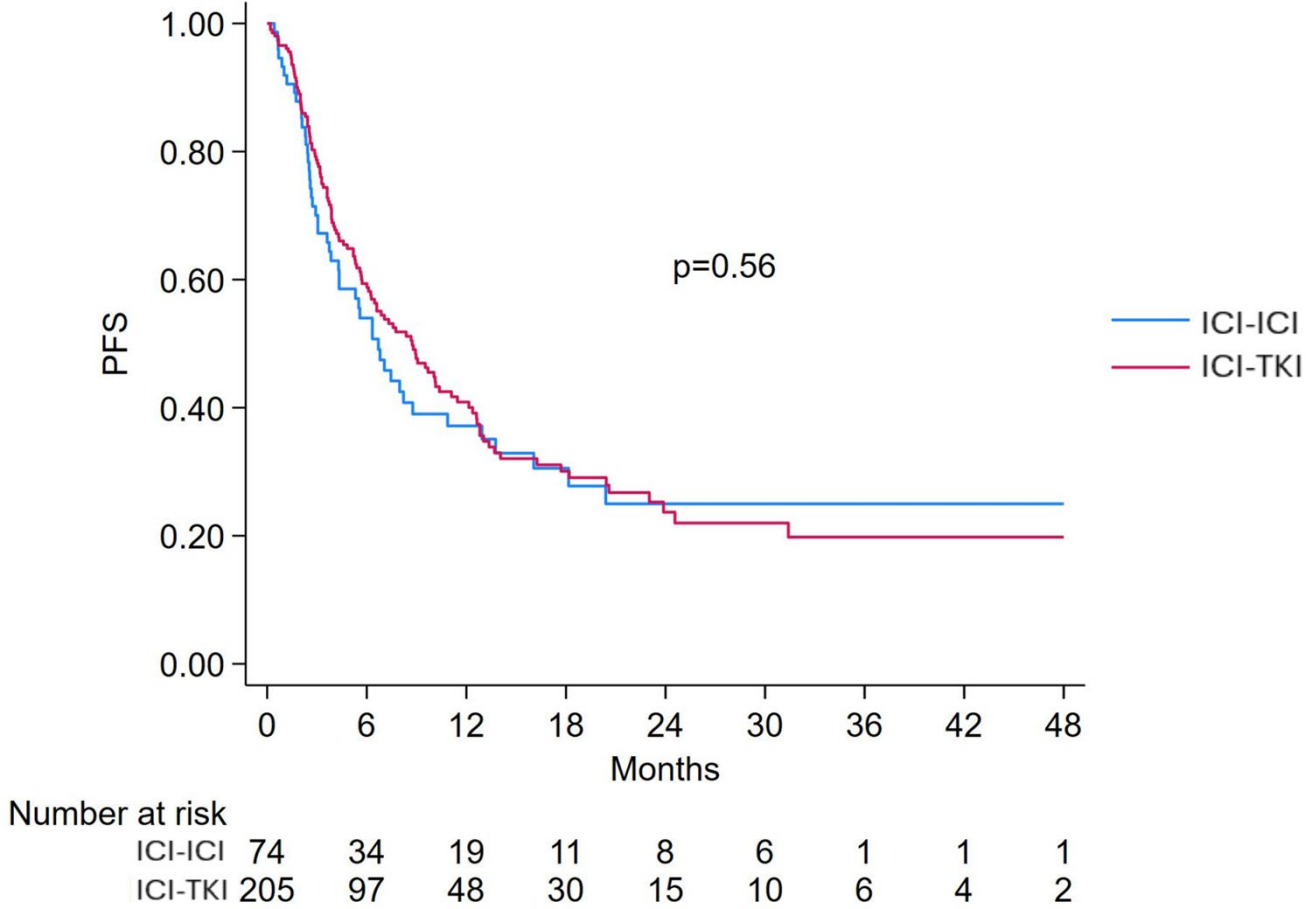
Progression-free survival in IMDC poor-risk patients treated with ICI-ICI versus ICI-TKI combinations. ICI: immune-checkpoint inhibitors; TKI: tyrosine-kinase inhibitors; PFS: Progression-free survival

PFS was evaluable in 297/311 poor-risk patients, 74 treated with ICI-ICI and 205 with ICI-TKI. The median PFS of the cohort was 7.8 months (95% CI 6.3–10.1). No significant difference (HR 1.10, 95% CI 0.79–1.54, *p* = 0.53) was observed between patients treated with ICI-ICI (6.7 months, 95% CI 4.3–10.9) and ICI-TKI (8.7 months, 95% CI 6.3–10.4), Fig. 3

**Fig. 4:**
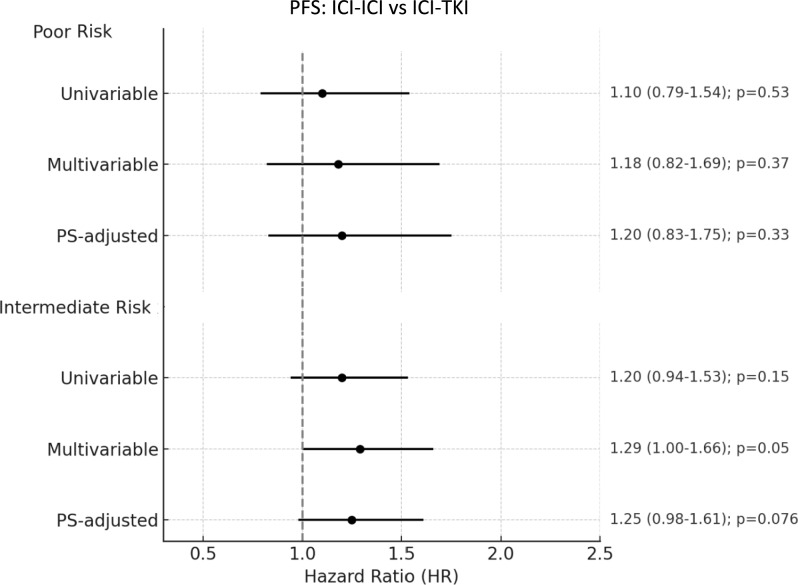
Forest plot of progression-free survival comparing ICI-ICI versus ICI-TKI combinations in IMDC poor- and intermediate-risk groups. ICI: immune-checkpoint inhibitors; TKI: tyrosine-kinase inhibitors; PFS: Progression-free survival; PS: propensity score

The multivariable model showed that presence of bone metastases (HR 1.53, 95% CI 1.12–2.10, *p* = 0.008) and sarcomatoid components (HR 1.58, 95% CI 1.04–2.38, *p* = 0.031) were significantly associated with shorter PFS, Table 2. Type of treatment (ICI-ICI vs. ICI-TKI) was not significantly associated with PFS (HR 1.18, 95% CI 0.82–1.69, *p* = 0.37), even when PS-adjusted (HR 1.20, 95% CI 0.83–1.75, *p* = 0.330), despite an overall trend favoring ICI-TKI, Fig. 4.

#### Objective response rate (ORR)

Among 229 evaluable patients, 63 in the ICI-ICI group and 166 in the ICI-TKI group, the ORR was 45.0%. Response rates were similar between groups: 42.9% in the ICI-ICI group and 45.8% in the ICI-TKI group, Supplementary Table 3.

**Fig. 5:**
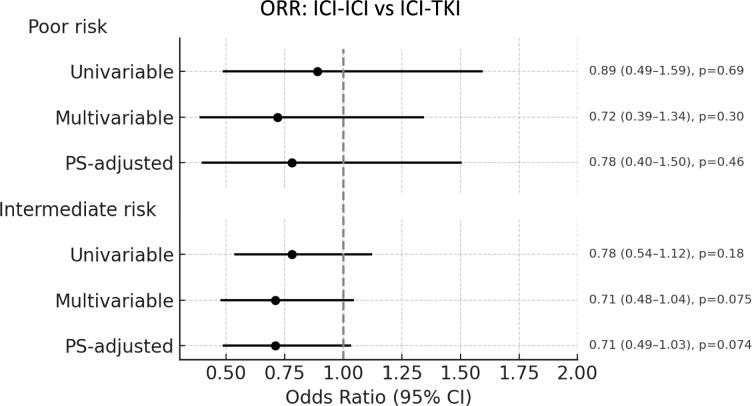
Forest plot of objective response rate (ORR) comparing ICI-ICI versus ICI-TKI combinations in IMDC poor- and intermediate-risk groups. ICI: immune-checkpoint inhibitors; TKI: tyrosine-kinase inhibitors; PS: propensity score

In the univariable analysis, no significant differences in response rates were observed with ICI-ICI versus ICI-TKI (OR 0.89, 95% CI 0.49–1.59; *p* = 0.69). In multivariable analysis, presence of baseline bone metastases was identified as a factor significantly associated with lower response rate (OR 0.43, 95% CI 0.25–0.75, *p* = 0.003), while the association between treatment group (ICI-ICI vs. ICI-TKI) and ORR remained non-significant (OR 0.72, 95% CI 0.39–1.34, *p* = 0.303), Supplementary Table 4. Results were consistent when using a PS–adjusted model (OR 0.78, 95% CI 0.40–1.50; *p* = 0.46), despite a trend suggesting inferior response with ICI-ICI, Fig. 5.

### IMDC intermediate-risk

#### Baseline characteristics

Among the 877 intermediate-risk patients, 122 (13.9%) patients were treated with TKI monotherapy, although these were excluded from the final analysis, and 556 (63.4%) had ICI-TKI combinations as first-line systemic therapies. Axitinib plus pembrolizumab (31.4%) represented the most frequently used ICI-TKI regimen. Ipilimumab plus nivolumab (IO+IO) was used in 199 (22.7%) patients, Supplementary Table 2.

The mean age was 65.7 years (SD 10.3), with a predominance of male patients (73.9%). Most patients had a good performance status, with 96.0% presenting an ECOG PS of 0 or 1. Comorbidities were reported in 79.2% of patients, most commonly cardiovascular (62.7%) and metabolic/endocrine (32.6%). A prior nephrectomy had been performed in 60.6% of patients, suggesting a relevant proportion of patients presenting with metachronous metastatic disease. Clear cell carcinoma remained the most prevalent histologic subtype (84.9%), followed by papillary (6.2%), Table 1.

Patients treated with ICI-ICI had more frequently sarcomatoid (19.1% vs. 10.1%, *p* = 0.001) and rhabdoid components (12.1% vs. 6.5%, *p* = 0.011), tumor necrosis (31.2% vs. 22.1%, *p* = 0.042) and lung metastases at baseline (61.8% vs. 53.8%, *P* = 0.05). They were also more likely to have metabolic/endocrine comorbidities (42.2% vs. 29.1%, *p* = 0.001) and undergone prior surgery (68.7% vs. 57.8%, *p* = 0.007). Conversely, patients treated with ICI-TKI more frequently presented with liver (16.4% vs. 6.5%, *p* = 0.001) and bone metastases (33.3% vs. 23.6%, *p* = 0.011). As in poor-risk subgroup, only 25% of patients received a second-line treatment. Patients who received ICI-TKI combinations as first-line treatment were more likely to have a second-line treatment (77.2% vs. 66.8%, p = 0.004).

#### Survival outcomes by treatment type

Median follow-up in the intermediate-risk subgroup was 14.5 months (IQR: 6.5–25.1). Among the 877 intermediate-risk patients, OS was evaluable in 810 patients (92.4%), including 187 treated with ICI-ICI and 512 with ICI-TKI.

**Fig. 6:**
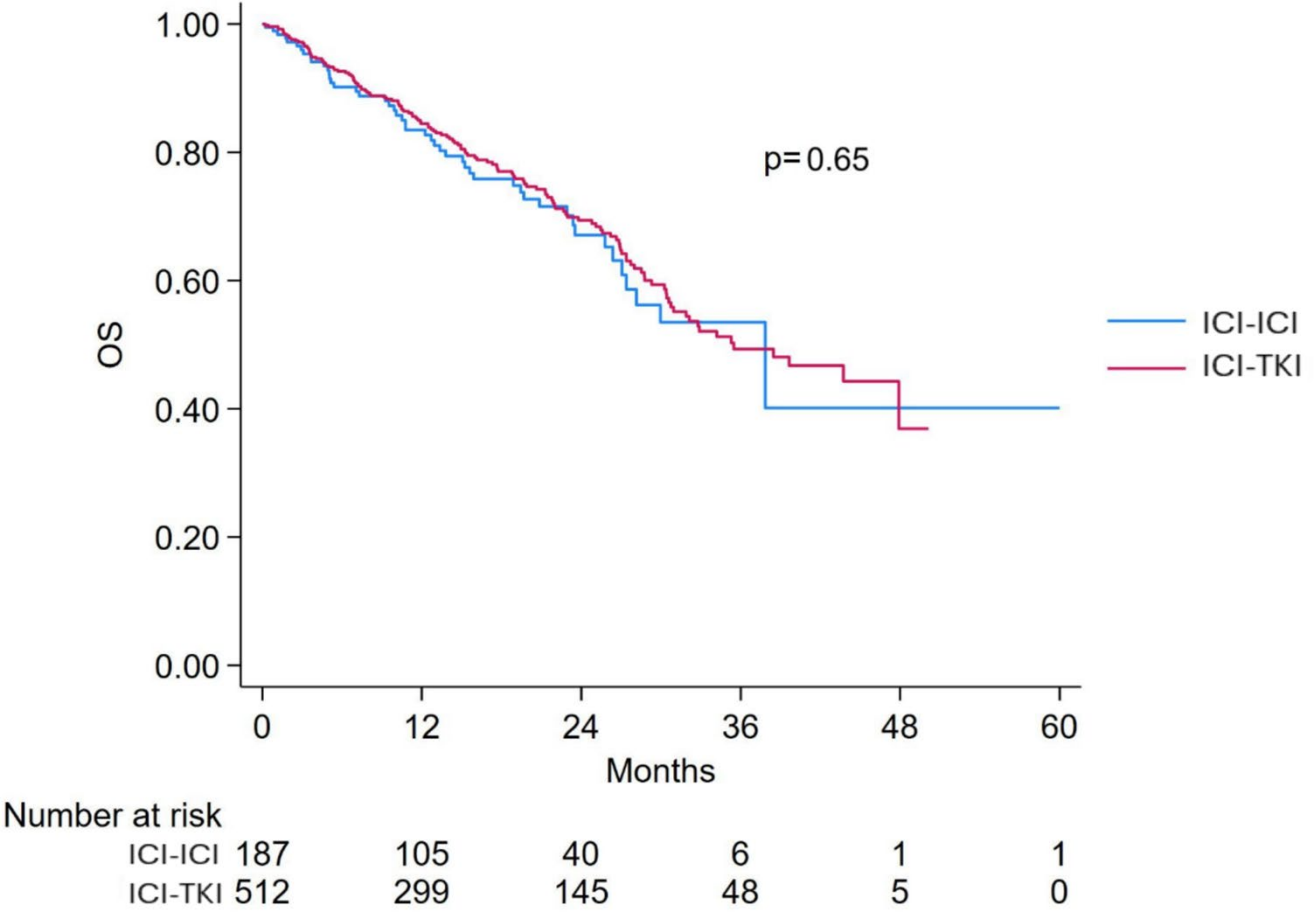
Overall survival in IMDC intermediate-risk patients treated with ICI-ICI versus ICI-TKI combinations. ICI: immune-checkpoint inhibitors; TKI: tyrosine-kinase inhibitors; OS: Overall survival

The median OS for the intermediate-risk population was 38.5 months (95% CI 32.9–NR), with similar values observed in the ICI-ICI group (37.8 months, 95% CI 27.1–NR) and the ICI-TKI group (35.5 months, 95% CI 30.6–NR), Fig. 6. In the univariable analysis, no significant difference in OS was observed between treatment groups (HR 1.08; 95% CI 0.77–1.50; *p* = 0.65), Fig. 2. Additionally, no significant interaction was detected between risk group (poor vs. intermediate) and treatment effect (*p* = 0.39).

In the multivariable model, factors independently associated with shorter OS were presence of bone (HR 1.58; 95% CI 1.14–2.19; *p* = 0.006), lung (HR 1.71; 95% CI 1.24–2.37; *p* = 0.001), and liver metastases (HR 2.97; 95% CI 2.10–4.19; *p* < 0.001), Table 3. The type of first-line treatment was not independently associated with OS (HR 1.21; 95% CI 0.86–1.69; *p* = 0.276), even when adjusting for PS model (HR 1.12; 95% CI 0.80–1.56; *p* = 0.52), Fig. 2.

**Table 3:** Multivariable cox regression analysis for overall survival and progression-free survival in IMDC intermediate-risk patients

Variable	Reference	Overall survival		Progression-free survival	
		HR (95% CI)	*p*-value	HR (95% CI)	*p*-value
Treatment ICI-ICI	ICI-TKI	1.21 (0.86–1.69)	0.276	1.29 (1.00–1.66)	0.050
Previous surgery	No	0.61 (0.45–0.83)	0.002	0.73 (0.58–0.92)	0.008
Papillary histology	Clear cell	1.10 (0.61–1.96)	0.760	1.36 (0.87–2.11)	0.173
Chromophobe	Clear cell	1.70 (0.62–4.64	0.303	1.33 (0.54–3.24)	0.537
Poorly differentiated	Clear cell	1.51 (0.60–3.79)	0.383	1.39 (0.70–2.75)	0.346
Rhabdoid features	No	1.91 (0.93–3.94)	0.08	–	–
Bone metastases	No	1.58 (1.14–2.19)	0.006	1.40 (1.09–1.80)	0.008
Lung metastases	No	1.71 (1.24–2.37)	0.001	1.54 (1.20–1.97)	0.001
Liver metastases	No	2.97 (2.10–4.19)	< 0.001	1.84 (1.37–2.48)	< 0.001

ICI: immune-checkpoint inhibitor; TKI: tyrosine-kinase inhibitors; HR Hazard ratio

**Fig. 7:**
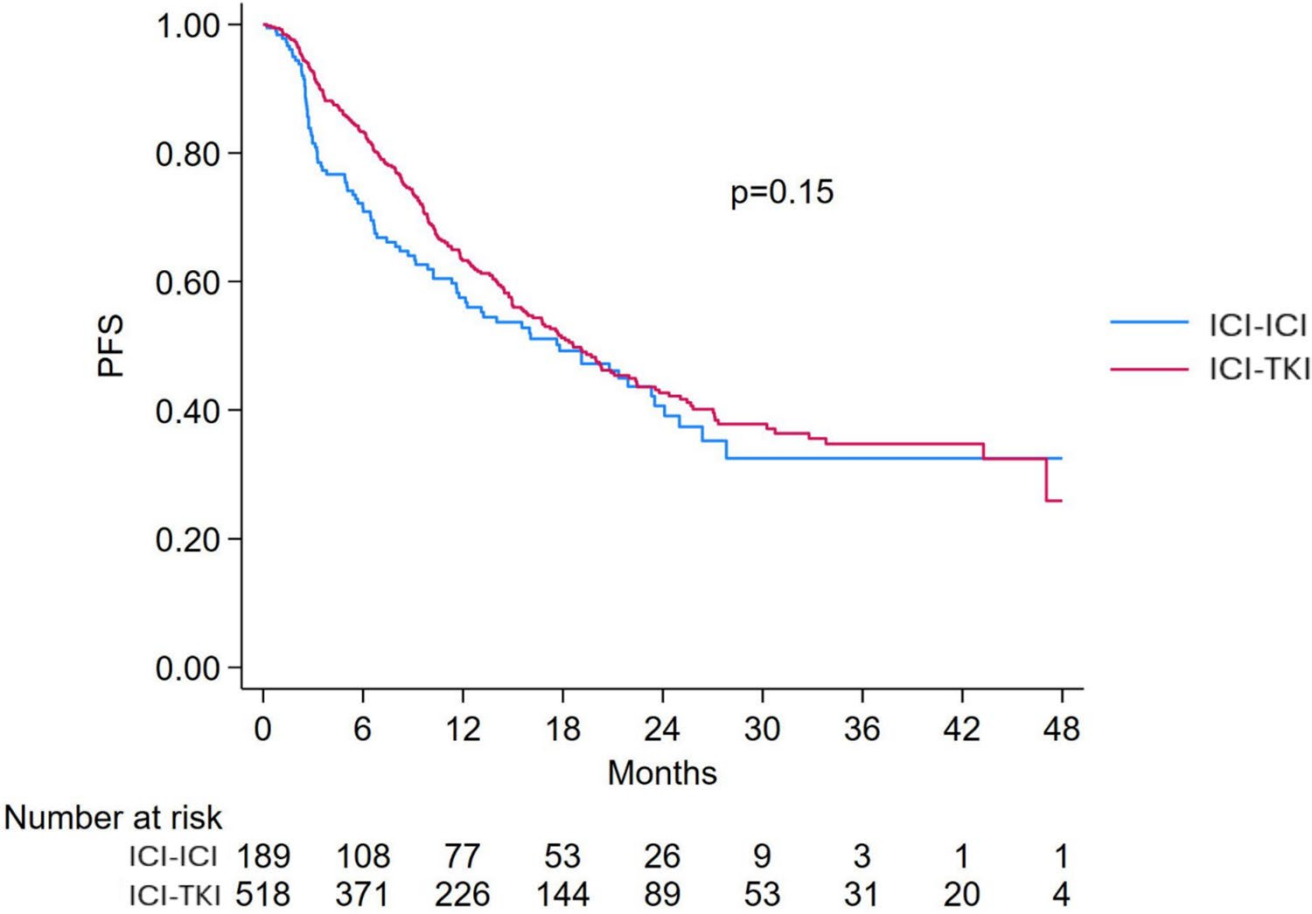
Progression-free survival in IMDC intermediate-risk patients treated with ICI-ICI versus ICI-TKI combinations. ICI: immune-checkpoint inhibitor; TKI: tyrosine-kinase inhibitors; PFS: Progression-free survival

PFS was evaluable in 611 out of 653 intermediate-risk patients, with 145 treated with ICI-ICI and 397 with ICI-TKI (total 542 patients). The median PFS for the intermediate-risk group was 17.9 months (95% CI 15.5–20.3), with similar medians for patients treated with ICI-ICI (17.8 months, 95% CI 11.7–23.5) and ICI-TKI (18.6 months, 95% CI 15.8–22.4), Fig. 7. No significant difference was found in the univariable analysis (HR 1.20; 95% CI 0.94–1.53; *p* = 0.15), and the interaction with risk group remained non-significant (*p* = 0.65), Fig. 4.

In the multivariable analysis, significant predictors of shorter PFS included the presence of bone (HR 1.40; 95% CI 1.09–1.80; *p* = 0.008), lung (HR 1.54; 95% CI 1.20–1.97; *p* = 0.001), and liver metastases (HR 1.84; 95% CI 1.37–2.48; *p* < 0.001). Prior surgery was associated with improved PFS (HR 0.73; 95% CI 0.58–0.92; *p* = 0.008), Table 3. Treatment with ICI-ICI was associated with a trend toward shorter PFS compared to ICI-TKI (HR 1.29; 95% CI 1.00–1.66; *p* = 0.050), although a PS-adjusted model confirmed no significant difference in PFS between treatment groups (HR 1.25; 95% CI 0.98–1.61; *p* = 0.076), Fig. 4.

#### Objective response rate

Among 618 evaluable intermediate-risk patients (154 in the ICI-ICI group and 464 in the ICI-TKI group), the ORR was 52.7%. Response rates were 48.0% in the ICI-ICI group and 54.3% in the ICI-TKI group, Supplementary Table 3. The treatment type (ICI-ICI vs. ICI-TKI) was not significantly associated with ORR in both the multivariable (OR 0.71, 95% CI 0.48–1.04; *p* = 0.075) and PS-adjusted models (OR: 0.71; 95% CI 0.49–1.03; *p* = 0.074), despite a trend favoring higher response rates with ICI-TKI combinations, Fig. 5. In the multivariable regression analysis, presence of lung metastases (OR 1.50, 95% CI 1.07–2.02, *p* = 0.018) was significantly associated with higher response rates. Conversely, presence of rhabdoid features (OR 0.53, 95% CI 0.29–0.98, *p* = 0.043) and baseline bone metastases (OR 0.46, 95% CI 0.23–0.94, *p* = 0.035) were associated with lower response rate, Supplementary Table 4.

#### Sensitivity analyses restricted to clear cell histology

Forest plots summarizing the results of the sensitivity analyses for OS, PFS and ORR in both IMDC poor- and intermediate-risk groups are reported in the Supplementary Fig. 2.

Overall, the direction and magnitude of treatment effects were consistent with those observed in the overall population. In poor-risk patients, no statistically significant differences between ICI–ICI and ICI–TKI were observed for OS, PFS or ORR across univariable, multivariable and PS–adjusted models, with wide confidence intervals, likely reflecting the limited sample size of this subgroup.

In intermediate-risk patients, ICI–TKI was associated with a significantly higher ORR across all modelsand with significantly longer PFS in the PS–adjusted analysis, while no significant differences in OS were observed. These findings likely reflect increased statistical power in the intermediate-risk subgroup rather than a qualitatively different treatment effect.

### Discussion

In the present sub-analysis of the MEET-URO33 study, we analysed real-world data from a large multicentre cohort of retrospective and prospective patients with mRCC classified as intermediate- or poor-risk according to the IMDC score and treated with first-line immune-based combinations (ICI-ICI and ICI-TKI). To our knowledge, this is among the largest retro- and prospective observational analyses conducted to date, aiming to investigate the comparative effectiveness of immune-based combinations in distinct IMDC subgroups.

We found that ICI-ICI combinations were preferentially administered to patients with sarcomatoid and rhabdoid features, due to their reduced sensitivity to TKIs, and multiple systemic comorbidities, given the known side effect profile of TKIs. Conversely, ICI-TKI regimens were often administered with features suggestive of high or symptomatic metastatic burden, including bone and liver involvement, likely with the aim of achieving a quicker response. Overall, in both intermediate- and poor-risk patients, ICI-TKI regimens were more frequently prescribed, although head-to-head comparisons between different immune-based combinations are limited. This likely reflects clinicians’ preferences in prescribing, based on their experience and subgroup analyses from randomized studies.

In this study, we found no statistically significant differences in PFS and OS between ICI-ICI and ICI-TKI regimens in either subgroup, even when adjusting for known prognostic factors and imbalanced baseline characteristics using a multivariable PS-adjusted model. Taken together, these findings suggest that the choice between ICI-ICI and ICI-TKI combinations may result in comparable outcomes in IMDC poor- and intermediate-risk patients, with no clear survival advantage associated with one strategy over the other. This also applies to poor-risk patients, for whom an ICI-TKI combination is often preferred due to its potentially greater ability to achieve an objective response. Instead, a trend favouring OS in poor-risk patients treated with ICI-ICI was observed, with an absolute difference of approximately 7 months. A numerical trend toward longer PFS was also observed with ICI-TKI combinations in both subgroups, more pronounced for intermediate-risk patients. The lack of statistical significance, despite numerically and clinically relevant differences, may reflect the underrepresentation of patients receiving ICI–ICI combinations (approximately 25%) across both intermediate- and poor-risk groups. An alternative interpretation regarding the lack of differences between ICI-ICI and ICI-TKI combinations may be that, when clinicians select treatment appropriately for each patient, the outcomes achieved in real-world practice may approach the best possible results for each subgroup. It is also worth noting that approximately 20% of patients in the poor-risk group and 15% in the intermediate-risk subgroup had a non–clear cell histology, which may have affected the results. When restricting the analysis to patients with clear cell histology, sensitivity analyses showed a longer PFS with ICI–TKI combinations in intermediate-risk patients, reaching statistical significance after PS-adjustment.

Interestingly, ORR was also comparable across treatment strategies in both risk groups. No significant association was found between treatment type and ORR in PS-adjusted models, although a consistent trend towards lower responses with ICI-ICI combinations emerged, again more pronounced for intermediate-risk patients. In the sensitivity analysis restricted to patients with clear cell histology, a significant advantage of ICI–TKI over ICI–ICI in terms of ORR among intermediate-risk patients was observed. This is likely due to greater statistical power rather than a qualitatively different treatment effect. Of note, a numerical benefit was also present in poor-risk patients, although the smaller sample size in this subgroup likely limited the statistical power. These findings may question the commonly held assumption that adding a TKI to immunotherapy necessarily increases the likelihood of achieving a response. That said, in clinical practice, what often drives treatment decisions is not just the occurrence of a response but its timing. The time to clinical response remains a crucial endpoint—especially in symptomatic patients or those with rapidly progressing disease where early tumor shrinkage is clinically relevant.

Overall, these results partially align with the prior ARON-1 retrospective multicentric study, where ICI-TKI combinations was clearly associated with better PFS and OS in intermediate-risk patients, while outcomes in poor-risk patients were not different across regimens [12]. Despite the similar sample size and median follow-up, the present study included a prospective cohort, as well as PS-score analysis and interaction test, which overall strengthen the results of our analysis. On the other hand, the ARON-1 had a higher proportion of patients treated with ICI-ICI in both poor- and intermediate-risk subgroups (approximately 50%), while in the present study only approximately 25% received ICI-ICI in both groups. The recent COSMIC-313 trial also reported a lack of OS benefit from adding cabozantinib to ipilimumab-nivolumab in both intermediate- and poor-risk patients, while demonstrating an improved PFS limited to intermediate-risk patients [13, 14]. In our study, the impact of the type of treatment was not significant across risk subgroups. In the pivotal trials of immune-based combinations for mRCC, all regimens were compared against sunitinib, and no head-to-head randomized comparisons between ICI-ICI and ICI-TKI combinations, either overall or divided by IMDC subgroups, are available. However, a recent work on the CheckMate 214 showed a consistent benefit of nivolumab plus ipilimumab in terms of ORR, OS and PFS across all intermediate- and poor-risk subgroups, regardless of the number of IMDC risk factors. Interestingly, ORR remained stable regardless of the number of IMDC risk factors, ranging from approximately 39–44%, with CR consistently observed across most subgroups. The ORR rates observed in our cohort closely reflects these evidence [15]. Moreover, a recent network meta-analysis integrating data from pivotal phase III trials of immune-based combinations showed that ICI–TKI regimens, particularly pembrolizumab plus lenvatinib, had the highest benefit in terms of PFS and ORR in both intermediate- and poor-risk patients, while nivolumab plus ipilimumab remains associated with long-term survival and the highest CR rates, despite lower ORR [16, 17]. These works also suggested that, among intermediate-risk patients, several ICI–TKI combinations achieved superior PFS compared with nivolumab plus ipilimumab, whereas differences were less pronounced in poor-risk groups [16, 17]. Our findings align with these data, in particular, the trend toward longer PFS and higher ORR with ICI–TKI combinations in intermediate-risk patients, becoming statistically significant in sensitivity analyses restricted to clear cell histology. On the other hand, the absence of a clear OS advantage for ICI–TKI over ICI-ICI, together with a numerical OS trend favoring ICI–ICI in poor-risk patients, may reflect the durable survival benefit observed with nivolumab plus ipilimumab in CheckMate 214 and subsequent post-hoc analyses [15]. These patterns may be explained by the biological mechanisms underlying IMDC risk groups: angiogenesis-related signatures are more prominent in favorable-risk disease and progressively less present in higher-risk tumors, whereas poor-risk RCC is characterized by a more inflamed tumor microenvironment. Intermediate-risk disease likely represents a mixed state, in which both angiogenic signature and immune dysfunction exist [18]. In this setting, the addition of a TKI may enhance tumor shrinkage, while dual immune checkpoint blockade may promote durable immune responses. This may also explain the recent results of the already mentioned COSMIC-313 trials [14].

Several limitations of our study should be acknowledged. First, despite robust statistical adjustment and the use of PS models, the predominant retrospective nature of the analysis cannot fully account for unmeasured confounders or selection bias that may have influenced treatment allocation and outcomes. Second, the relatively short follow-up time, particularly among poor-risk patients, and the high number of censored patients, may limit our ability to capture long-term survival differences and fully assess the durability of treatment benefit. This is especially relevant with immune-based combinations, where long-term survival and delayed treatment effects may emerge over extended follow-up. A longer follow-up of the study population, along with continued accrual of the prospective component of the study, will be crucial to more accurately assess long-term survival and to strengthen our findings. A further limitation is the smaller number of patients and shorter follow-up available for the latest combinations approved, particularly pembrolizumab plus lenvatinib. Indeed, pembrolizumab and axitinib was the most prescribed combination with the greater follow-up period. This may limit the generalizability of our findings, reducing the comprehensiveness in representing the current treatment landscape. Again, the ongoing recruitment and prospective expansion of the study will allow for a more complete evaluation of the newer combinations. As previously mentioned, the population treated with ICI-cxsICI was underrepresented (approximately 25%), limiting statistical power and the generalizability of direct comparisons with ICI–TKI regimens: this may have contributed to the lack of statistically significant differences across some endpoints., Furthermore, the short follow-up might have impacted on the low number of patients who received a second-line treatment. Moreover, although PS-adjustment was used to mitigate baseline imbalances between treatment groups, this approach cannot eliminate residual confounding and treatment-selection bias, particularly in the presence of unmeasured or incompletely captured clinical factors. Therefore, the absence of statistically significant differences between treatment strategies should be interpreted with caution and should not be considered evidence of true therapeutic equivalence. Eventually, an additional limitation is the lack of detailed information on TKI dose intensity: dose reductions and treatment interruptions over time may have influenced efficacy outcomes further confounding the interpretability of our results.

### Conclusion

In this large real-world cohort of intermediate- and poor-risk mRCC patients treated with first-line immune-based combinations, no significant differences in survival or response rates were observed between ICI-ICI and ICI-TKI regimens. These findings support the use of current clinical and histological criteria as tools to guide the choice of first-line combination therapy. At the same time, they underscore the need for refined clinical and molecular tools to optimize treatment selection in mRCC and warrant prospective validation in risk-tailored clinical trials.

## Supplementary Information

Below is the link to the electronic supplementary material.

Supplementary file1 (DOCX 422 kb)

## Data Availability

The datasets generated during and/or analysed during the current study are available from the corresponding author on reasonable request.
