# Supplementary material for: Effectiveness of ICI-ICI versus ICI-TKI combinations in patients with IMDC intermediate- and poor-risk metastatic renal cell carcinoma: a sub-analysis of the MEET-URO 33 study
Source: Cancer Immunol Immunother. 2026 Feb 3;75(3):66. doi: 10.1007/s00262-026-04318-x (PMC12868551; doi:10.1007/s00262-026-04318-x)

**Supplementary Fig.1** Consort diagram of patient and treatment distribution by IMDC risk group.


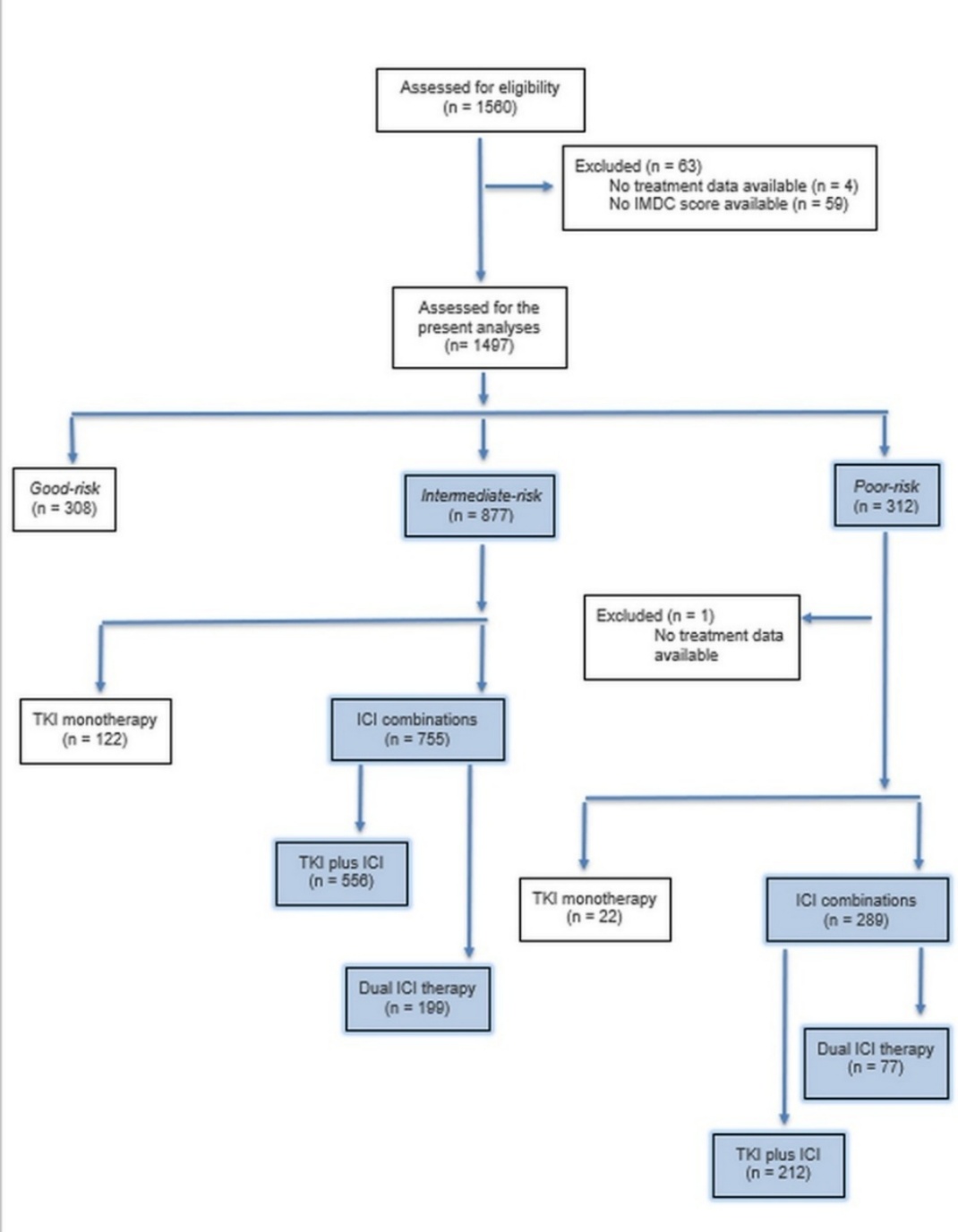


Abbreviations: TKI: tyrosine-kinase inhibitors; ICI: Immune-checkpoint inhibitor.

**Supplementary Table 1.** Baseline characteristics of the overall population by IMDC group.

|  | | POOR-RISK | | | | INTERMEDIATE-RISK | | | |
| --- | --- | --- | --- | --- | --- | --- | --- | --- | --- |
| Variable | Category | Total  n = 289  n (%) | ICI-ICI  n = 77  n (%) | ICI-TKI  n = 212  n (%) | *p*-value | Total  n = 755  n (%) | ICI-ICI  n = 199  n (%) | ICI-TKI  n = 556  n (%) | *p*-value |
| Age  mean (SD) |  | 64.3  (10.8) | 66.4  (11.4) | 63.5  (10.5) | 0.056 |  | 65.7  (10.3) | 64.4  (10.8) | 0.14 |
| Gender | Females | 82  (28.4) | 22  (28.6) | 60  (28.3) | 0.99 | 196/754 (26.0) | 47  (23.6) | 149/555 (26.8) | 0.37 |
|  | Males | 207  (71.6) | 55  (71.4) | 152  (71.7) |  | 558 | 152  (76.4) | 406  (73.2) |  |
| ECOG | 0 | 101  (34.9) | 29  (37.7) | 72  (34.0) | 0.83 | 513/754 (68.0) | 144  (72.4) | 369  (66.5) | 0.33 |
|  | 1 | 121  (41.9) | 29  (37.7) | 92  (43.4) |  | 211  (28.0) | 50  (25.1) | 161  (29.0) |  |
|  | 2 | 58  (20.1) | 16  (20.8) | 42  (19.8) |  | 27  (3.6) | 4  (2.0) | 23  (4.1) |  |
|  | 3 | 9  (3.1) | 3  (3.9) | 6  (2.8) |  | 3  (0.4) | 1  (0.5) | 2  (0.4) |  |
| Comorbidities | Presence | 233  (74.9) | 64  (83.1) | 152  (71.7) | 0.065 | 598  (79.2) | 165  (82.9) | 433  (77.9) | 0.13 |
| Cardiovascular comorbidity | Presence | 177  (61.2) | 58  (75.3) | 119  (56.1) | **0.004** | 473  (62.7) | 132  (66.3) | 341  (61.3) | 0.21 |
| Dermatologic comorbidity | Presence | 6  (2.1) | 4  (5.2) | 2  (0.9) | **0.045** | 18  (2.4) | 6  (3.0) | 12  (2.2) | 0.50 |
| Hematologic comorbidity | Presence | 19  (6.6) | 4  (5.2) | 15  (7.1) | 0.789 | 40  (5.3) | 9  (4.5) | 31  (5.6) | 0.57 |
| Gastrointestinal comorbidity | Presence | 23  (8.0) | 6  (7.8) | 17  (8.0) | 0.99 | 64  (8.5) | 18  (9.1) | 46  (8.3) | 0.74 |
| Genitourinary comorbidity | Presence | 31  (10.7) | 15  (19.5) | 16  (7.5) | **0.008** | 110  (14.6) | 31  (15.6) | 79  (14.2) | 0.64 |
| Metabolic or endocrine comorbidity | Presence | 84  (29.1) | 28  (36.4) | 56  (26.4) | 0.11 | 246  (32.6) | 84  (42.2) | 162  (29.1) | **0.001** |
| Neurologic comorbidity | Presence | 23  (8.0) | 10  (13.0) | 13  (6.1) | 0.082 | 53  (7.0) | 14  (7.0) | 39  (7.0) | 0.99 |
| Sensory organ comorbidity | Presence | 14  (4.8) | 5  (6.5) | 9  (4.2) | 0.54 | 23  (3.1) | 8  (4.0) | 15  (2.7) | 0.35 |
| Osteoarticular comorbidity | Presence | 10  (3.5) | 3  (3.9) | 7  (3.3) | 0.73 | 42  (5.6) | 10  (5.0) | 32  (5.8) | 0.70 |
| Respiratory comorbidity | Presence | 22  (7.6) | 10  (13.0) | 12  (5.7) | **0.046** | 44  (5.8) | 17  (8.5) | 27  (4.9) | 0.057 |
| Concomitant drugs | Yes | 232/288 (80.6) | 68  (88.3) | 164/211 (77.7) | **0.045** | 571  (75.6) | 157  (78.9) | 414  (74.5) | 0.352 |
| Low dose steroids | Yes | 33/288 (11.5) | 9  (11.7) | 24/211 (11.4) | 0.99 | 48/751 (6.4) | 13/199 (6.5) | 35/552 (6.3) | 0.92 |
| High dose steroids | Yes | 39/288 (13.5) | 12  (15.6) | 27/211 (12.8) | 0.56 | 43/750 (5.7) | 11/199 (5.5) | 32/551 (5.8) | 0.88 |
| Surgery | Yes | 106  (36.7) | 31  (40.3) | 75  (35.4) | 0.49 | 456/752 (60.6) | 136/198 (68.7) | 320/554 (57.8) | **0.007** |
| Nephrectomy | Partial | 16/106 (15.1) | 4/31  (12.9) | 12/75 (16.0) | 0.70 | 44/454 (5.8) | 8/136  (4.0) | 36/318 (6.5) | 0.165 |
|  | Radical | 84/106 (79.2) | 26/31 (83.9) | 58/75 (77.3) |  | 383  (50.7) | 121  (60.8) | 262  (47.1) |  |
|  | Tumorectomy | 6/106  (5.7) | 1/31  (3.2) | 5/75  (6.7) |  | 27  (3.6) | 7  (3.5) | 20  (3.6) |  |
| Stage T | 0 | 2/275  (0.7) | 0/75  (0.0) | 2/200  (1.0) | 0.52 | 2  (0.3) | 1  (0.5) | 1  (0.2) | **0.006** |
|  | 1 | 38/275 (13.8) | 11/75 (14.7) | 27/200 (13.5) |  | 131  (17.4) | 26  (13.1) | 105  (18.9) |  |
|  | 2 | 28/275 (10.2) | 6/75  (8.0) | 22/200 (11.0) |  | 129  (17.1) | 32  (16.1) | 97  (17.4) |  |
|  | 3 | 106/275 (38.5) | 35/75 (46.7) | 71/200 (35.5) |  | 314  (41.6) | 105  (52.8) | 209  (37.6) |  |
|  | 4 | 54/275 (19.6) | 13/75 (17.3) | 41/200 (20.5) |  | 62  (8.2) | 10  (5.0) | 52  (9.4) |  |
|  | X | 47/275 (17.1) | 10/75 (13.3) | 37/200 (18.5) |  | 86  (11.4) | 18  (9.0) | 68  (12.2) |  |
| Stage N | 0 | 82/276 (29.7) | 20/75 (26.7) | 62/201 (30.8) | 0.84 | 254  (33.6) | 78  (39.2) | 176  (31.7) | 0.382 |
|  | 1 | 57/276 (20.7) | 18/75 (24.0) | 39/201 (19.4) |  | 128  (17.0) | 34  (17.1) | 94  (16.9) |  |
|  | 2 | 29/276 (10.5) | 7/75  (9.3) | 22/201 (10.9) |  | 42  (5.6) | 8  (4.0) | 34  (6.1) |  |
|  | 3 | 6/276  (2.2) | 1/75  (1.3) | 5/201  (2.5) |  | 6  (0.8) | 2  (1.0) | 4  (0.7) |  |
|  | X | 102/276 (37.0) | 29/75 (38.7) | 73/201 (36.3) |  | 286  (37.9) | 70  (35.2) | 216  (38.8) |  |
| Stage M | 0 | 55/282 (19.5) | 16/76 (21.1) | 39/206 (18.9) | 0.846 | 265  (35.1) | 86  (43.2) | 179  (32.2) | **0.026** |
|  | 1 | 209/282 (74.1) | 56/76 (73.7) | 153/206 (74.3) |  | 376  (49.8) | 86  (43.2) | 290  (52.2) |  |
|  | X | 18/282 (6.4) | 4/76  (5.3) | 14/206 (6.8) |  | 74  (9.8) | 19  (9.5) | 55  (9.9) |  |
| Histology | Clear cell | 232  (80.3) | 62  (80.5) | 170  (80.2) | 0.844 | 641  (84.9) | 179  (89.9) | 462  (83.1) | **0.024** |
|  | Chromophobe | 3  (1.0) | 0  (0.0) | 3  (1.4) |  | 11  (1.5) | 1  (0.5) | 10  (1.8) |  |
|  | Undifferentiated | 26  (9.0) | 8  (10.4) | 18  (8.5) |  | 20  (2.6) | 7  (3.5) | 13  (2.3) |  |
|  | Papillary | 15  (5.2) | 4  (5.2) | 11  (5.2) |  | 47  (6.2) | 4  (2.0) | 43  (7.7) |  |
|  | Other | 13  (4.5) | 3  (3.9) | 10  (4.7) |  | 31  (4.1) | 7  (3.5) | 24  (4.3) |  |
| Sarcomatoid features | Yes | 47/287 (16.4) | 22  (28.6) | 25/210 (11.9) | **0.001** | 94/749 (12.5) | 38/197 (19.1) | 56/552 (10.1) | **0.001** |
| Rhabdoid features | Yes | 22/287 (7.7) | 10  (13.0) | 12/210 (5.7) | **0.012** | 60/747 (7.9) | 24/197 (12.1) | 36/550 (6.5) | **0.011** |
| Genomic signature | Yes | 22/284 (7.7) | 7  (9.1) | 15/207 (7.2) | 0.621 | 31/731 (4.1) | 9/196  (4.5) | 22/535 (4.0) | 0.94 |
| Necrosis | Yes | 82/215 (38.1) | 25/61 (41.0) | 57/154 (37.0) | 0.641 | 185  (24.5) | 62  (31.2) | 123  (22.1) | **0.042** |
| Lung metastases | Presence | 194  (67.1) | 49  (63.6) | 145  (68.4) | 0.48 | 422  (55.9) | 123  (61.8) | 299  (53.8) | **0.050** |
| Lymph-node metastases | Presence | 157  (54.3) | 44  (57.1) | 113  (53.3) | 0.60 | 329  (43.6) | 92  (46.2) | 237  (42.6) | 0.38 |
| Liver metastases | Presence | 55  (19.0) | 13  (16.9) | 42  (19.8) | 0.62 | 104  (13.8) | 13  (6.5) | 91  (16.4) | **0.001** |
| Bone metastases | Presence | 131  (45.3) | 22  (28.6) | 109  (51.4) | **0.001** | 232  (30.7) | 47  (23.6) | 185  (33.3) | **0.011** |
| Adrenal metastases | Presence | 52  (18.0) | 14  (18.2) | 38  (17.9) | 0.99 | 90  (11.9) | 27  (13.6) | 63  (11.3) | 0.40 |
| Brain metastases | Presence | 21  (7.3) | 4  (5.2) | 17  (8.0) | 0.61 | 42  (5.6) | 15  (7.5) | 27  (4.9) | 0.16 |
| Pancreatic metastases | Presence | 7  (2.4) | 1  (1.3) | 6  (2.8) | 0.68 | 55 (7.3) | 6  (3.0) | 49  (8.8) | **0.007** |
| Thyroid metastases | Presence | 1  (0.3) | 0  (0.0) | 1  (0.5) | 0.99 | 2  (0.3) | 1  (0.2) | 1  (0.5) | 0.45 |
| Soft tissue metastases | Presence | 26  (9.0) | 12  (15.6) | 14  (6.6) | **0.033** | 61  (8.1) | 9  (4.5) | 52  (9.4) | **0.032** |
| Metastasectomy before 1^st^-line | Yes | 25  (8.7) | 7  (9.1) | 18  (8.5) | 0.817 | 92  (12.2) | 27  (13.6) | 65  (11.7) | 0.60 |
| Radical radiotherap*y* | Yes | 21  (7.3) | 2  (2.6) | 19  (9.0) | 0.075 | 49  (6.5) | 12  (6.0) | 37  (6.7) | 0.88 |
| Lung involvement at baseline | Presence | 206  (71.3) | 53  (68.8) | 153  (72.2) | 0.66 | 426  (56.4) | 125  (62.8) | 301  (54.1) | **0.042** |
| Lymph-node involvement baseline | Presence | 165  (57.1) | 47  (61.0) | 118  (55.7) | 0.42 | 344  (45.6) | 94  (47.2) | 250  (45.0) | 0.639 |
| Liver involvement at baseline | Presence | 62  (21.5) | 16  (20.8) | 46  (21.7) | 0.99 | 107  (14.2) | 15  (7.5) | 92  (16.5) | **0.003** |
| Bone involvement at baseline | Presence | 138  (47.8) | 23  (29.9) | 115  (54.2) | **<0.001** | 241  (31.9) | 47  (23.6) | 194  (34.9) | **0.005** |
| Adrenal involvement at baseline | Presence | 51  (17.6) | 13  (16.9) | 38  (17.9) | 0.99 | 89  (11.8) | 21  (10.6) | 68  (12.2) | 0.616 |
| Brain involvement at baseline | Presence | 23  (8.0) | 4  (5.2) | 19  (9.0) | 0.46 | 42  (5.6) | 16  (8.0) | 26  (4.7) | 0.110 |
| Pancreas involvement baseline | Presence | 6  (2.1) | 1  (1.3) | 5  (2.4) | 0.99 | 56  (7.4) | 6  (3.0) | 50  (9.0) | **0.009** |
| Thyroid involvement baseline | Presence | 1  (0.3) | 0  (0.0) | 1  (0.5) | 0.99 | 2  (0.3) | 1  (0.5) | 1  (0.2) | 1.000 |
| Soft tissue involvement baseline | Presence | 25  (8.7) | 11  (14.3) | 14  (6.6) | 0.056 | 58  (7.7) | 9  (4.5) | 49  (8.8) | 0.073 |
| Peritoneum involvement baseline | Presence | 4  (1.4) | 1  (1.3) | 3  (1.4) | 0.99 | 12  (1.6) | 4  (2.0) | 8  (1.4) | 0.824 |
| Other involvement baseline | Presence | 64  (22.1) | 23  (29.9) | 41  (19.3) | 0.077 | 426  (56.4) | 125  (62.8) | 301  (54.1) | **0.042** |
| 2^nd^-line thera*p*y | No | 204/267 (76.4) | 49/70 (70.0) | 155/197 (78.7) | 0.14 | 562  (74.4) | 133  (66.8) | 429  (77.2) | **0.004** |
|  | Yes | 63/267 (23.6) | 21/70 (30.0) | 42/197 (21.3) |  | 193  (25.6) | 66  (33.2) | 127  (22.8) |  |
| Type of 2^nd^-line | Combination | 7/63  (11.1) | 2/21  (9.5) | 5/42  (11.9) | 0.99 | 13  (1.7) | 3  (1.5) | 10  (1.8) | **0.027** |
|  | TKI | 56/63 (88.9) | 19/21 (90.5) | 37/42 (88.1) |  | 174  (23.0) | 58  (29.1) | 116  (20.9) |  |
|  | ICI | - | - | - |  | 6  (0.8) | 5  (2.5) | 1  (0.2) |  |
| Meet-Uro score | 2.0 | 169/259 (65.3) | 52/70 (74.3) | 117/189 (61.9) | 0.078 | 284  (37.6) | 83  (41.7) | 201  (36.2) | 0.075 |
|  | 3.0 | 90/259 (34.7) | 18/70 (25.7) | 72/189 (38.1) |  | 243  (32.2) | 68  (34.2) | 175  (31.5) |  |
|  | 4.0 | - | - | - |  | 110  (14.6) | 20  (10.1) | 90  (16.2) |  |

Abbreviations: TKI: tyrosine-kinase inhibitors; ICI: Immune-check*p*oint inhibitor; ECOG: Eastern Coo*p*erative Oncology Grou*p*; IMDC: International Metastatic RCC Database Consortium.

**Supplementary Table 2**. First-line treatment distribution by IMDC group.

|  |  | POOR-RISK | | INTERMEDIATE-RISK | |
| --- | --- | --- | --- | --- | --- |
| Therapy class | Therapy regimen | Frequency  (n = 311) | Percentage  (%) | Frequency  (n = 877) | Percentage (%) |
| ICI+ICI | Ipilimumab + Nivolumab | **77** | **24.8** | **199** | **22.7** |
| ICI+TKI |  | **212** | **68.2** | **556** | **63.4** |
|  | Axitinib + Pembrolizumab | 87 | 28.0 | 275 | 31.4 |
|  | Cabozantinib + Nivolumab | 85 | 27.3 | 172 | 19.6 |
|  | Lenvatinib + Pembrolizumab | 40 | 12.9 | 109 | 12.4 |
| TKI* |  | **22** | **7.1** | **122** | **13.9** |
|  | Sunitinib | 5 | 1.6 | 36 | 4.1 |
|  | Pazopanib | 5 | 1.6 | 30 | 3.4 |
|  | Cabozantinib | 12 | 3.9 | 36 | 4.1 |
| Other* | - | - | - | **20** | **2.3** |

Abbreviations: ICI: Immune-check*p*oint inhibitor; TKI: tyrosine-kinase inhibitors.*Excluded from final analysis.

**Supplementary Table 3.** Objective response rate (ORR) in IMDC poor- and intermediate-risk patients by treatment type.

|  | POOR-RISK | INTERMEDIATE-RISK |
| --- | --- | --- |
| Treatment group | **ORR**  n = 229  n (%) | **ORR**  n = 618  n (%) |
| ICI-ICI | 27 (42.9) | 74 (48.0) |
| ICI-TKI | 76 (45.8) | 252 (54.3) |
| Total | 103 (45.0) | 326 (52.7) |

Abbreviations: ICI: immune-checkpoint inhibitors; TKI: tyrosine-kinase inhibitors.

**Supplementary Table 4.** Logistic regression analysis for objective response rate in IMDC poor- and intermediate-risk patients

|  |  | POOR-RISK | | INTERMEDIATE-RISK | |
| --- | --- | --- | --- | --- | --- |
| Variable | **Reference** | **OR (95% CI)** | **p-value** | **OR (95% CI)** | **p-value** |
| Treatment ICI-ICI | ICI-TKI | 0.72 (0.39–1.34) | 0.303 | 0.71 (0.48 – 1.03) | 0.075 |
| Bone metastases | No | 0.43 (0.25–0.75) | **0.003** | 0.46 (0.23 – 0.94) | **0.035** |
| Lung metastases | No | - | - | 1.50 (1.07 – 2.02) | **0.018** |
| Rhabdoid features | No | - | - | 0.53 (0.29 – 0.98) | **0.043** |

Abbreviations: ICI: immune-checkpoint inhibitors; TKI: tyrosine-kinase inhibitors; OR: odds ratio.

**Supplementary Fig. 2** Forest plots for sensitivity analyses of OS (A), PFS (B) and ORR (C) restricted to patients with clear cell histology, stratified by IMDC intermediate- and poor risk groups


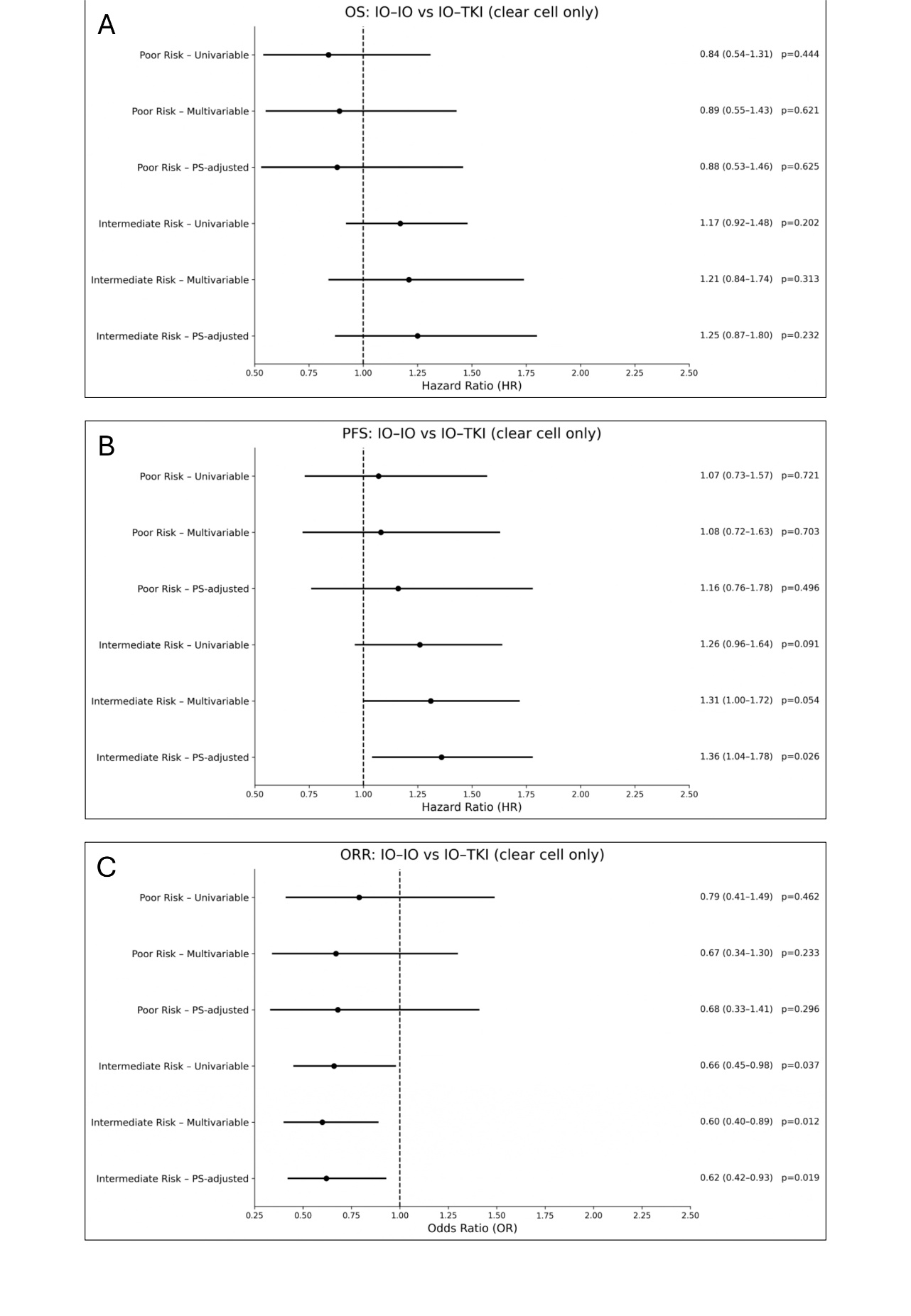

Supplement: Supplementary file 1 — Supplementary file1 (DOCX 422 kb) [file 262_2026_4318_MOESM1_ESM.docx]
